# Gender and age affect the levels of exhaled nitric oxide in healthy children

**DOI:** 10.3892/etm.2013.922

**Published:** 2013-01-23

**Authors:** HAN ZHANG, LINHUA SHU, XUXU CAI, ZHIJIA WANG, XUYONG JIAO, FEN LIU, PING HOU, LI WANG, LISHEN SHAN, NING CHEN, YUNXIAO SHANG

**Affiliations:** Department of Pediatrics, Shengjing Hospital of China Medical University, Shenyang, Liaoning 110004, P.R. China

**Keywords:** exhaled nitric oxide, healthy children, gender, age

## Abstract

Asthma is a chronic inflammatory disorder of the lung and diagnosis is difficult in children. The measurement of fractional exhaled nitric oxide (FeNO) may be useful in the diagnosis and monitoring of treatments. A number of factors affect FeNO levels and their influence varies across countries and regions. This study included 300 healthy students, aged from 6 to 14 years, who participated voluntarily. A comprehensive medical survey was used and measurements of FeNO levels and spirometric parameters were recorded in Shenyang, China. We observed that the median FeNO was 11 ppb (range, 8–16 ppb) in children from the northern areas of China. For males, the median level was 13 ppb (range, 9–18 ppb) and the median level was 10 ppb (range, 8–14 ppb) for females. There was a significant difference between males and females (P= 0.007) and age was correlated with FeNO (R^2^= 0.6554), while weight, height, body mass index (BMI), forced vital capacity (FVC), forced expiratory volume (FEV1), FEV1/FVC and peak expiratory flow (PEF) had no correlation with FeNO. In conclusion, the median FeNO is 11 ppb (range, 8–16 ppb) in male and female healthy children from northern areas of China and is affected by gender and age.

## Introduction

Fractional exhaled nitric oxide (FeNO) has been proposed as a biomarker of airway inflammation in asthma (1). The use of FeNO measurement in the diagnosis and monitoring of asthma necessitates the identification of reference values for FeNO measured with commercially available equipment (2). However, reference values vary between countries. Studies have shown that race is one of the most important variables when considering reference values (3) and the values from Caucasian children cannot be applied to Asian individuals (2–4). Currently, FeNO reference values exist for healthy Asian children in the southern areas of Hong Kong and Taiwan (3,4). This study aimed to establish FeNO reference values for the northern areas of China to supplement the existing body of research on Asian individuals.

## Materials and methods

### Study subjects

The participants included 300 healthy students recruited randomly from two primary schools and one middle school in Shenyang, China. The sample contained males and females, aged 6–14 years. The students were invited by the International Study of Asthma and Allergic Disease in Children (ISAAC) to complete a questionnaire (5). The subjects had no reported history of wheezing or whistling in the chest at any time in the past, including during or after exercise. Additionally, the subjects had no reported history of nasal (6,7) or asthmatic (8) problems and no history of dry cough at night, apart from a cough associated with a typical cold or chest infection in the last month. Moreover, any subject who in the previous two weeks had any episode of infection, active or passive exposure to cigarettes or parental or sibling disposition to asthma or atopy, was excluded. The parents of the subjects provided the relevant medical history information. The study was approved by the local Education Bureau and by the schools. Written informed consent was provided by parents and the subjects gave consent when aged 10 years or older. Forty-two subjects were excluded based on parental responses to the medical history questions. A total of 258 students participated in the FeNO meaurement and pulmonary function portions of the study following the survey.

### Measurements of FeNO

Testing occurred for 3 h in the morning. The temperature of the test room was fixed at 22–24°C. Single-breath online measurements of FeNO were taken by the Nitric Oxide Monitoring System (NIOX) according to the American Thoracic Society (ATS) guidelines (9–11). All subjects were asked not to eat, drink or participate in heavy exercise during the hour before testing. Testing began with the subject quiet and standing comfortably. First, the subject’s nose was clipped and the subject fully expelled air from the lungs, after which the subject inserted the mouthpiece of the NIOX system and inhaled NO-free air calmly to total lung capacity over a period of 2–3 sec. The subject then exhaled steadily, maintaining a constant air speed. If the subject breathed correctly, the machine emitted a continuous humming sound. Subjects exhaled at a flow rate of 50 ml/sec. Children ≥130 cm in height used a standard card to exhale for 10 sec while children ≤130 cm in height used a special card to exhale for 6 sec. The system automatically calculated the value during the last 3 sec of exhalation and displayed the value on the screen. Each subject repeated the measurements at least 3 times to obtain 3 reliable FeNO values, defined as three values varying by <10% from each other or two values varying by <5%. The means of the three values were then calculated. The subjects had a 2-min rest between individual FeNO measurements.

### Measurements of pulmonary function

Spirometry parameters for all subjects were measured using a Power-Cube Spirometer (Ganshorn, Niederlauer, Germany) according to the ATS guidelines (12). The guidelines included the use of nose clips and the adoption of a standing position after measurements of FeNO were taken. We received the predicted values of lung function variables, including forced vital capacity (FVC), forced expiratory volume (FEV1), FEV1/FVC and peak expiratory flow (PEF). If the FEV1 for a subject was <80%, the subject was excluded from further analyses. Values from 219 students were used.

### Measurements of body mass index (BMI)

The height and weight of the subjects were also recorded at the time of FeNO measurement.

### Statistical analysis

Data were analyzed with SPSS version 13.5 (SPSS Inc., Chicago, IL, USA). Due to the skewed distribution of FeNO values, the FeNO levels in parts per billion (ppb) were presented as the mean and median with interquartile range (IQR). Logarithmic transformations were calculated for the FeNO values. The results were presented using geometric means and upper 95% confidence limits. Either the Student’s t-test or the Mann-Whitney U test was used for between-group comparison. Since spirometry values (FVC, FEV1, FEV1/FVC and PEF) were not normally distributed, the arithmetic means were not used for further data analysis. Polynomial linear trend analysis was used to examine the correlation between FeNO and gender, age, weight, height, BMI, FVC, FEV1, FEV1/FVC and PEF. Linear regression, multivariate analysis and Pearson correlation analysis were also employed for this study. P<0.05 was considered to indicate a statistically significant difference.

## Results

**Figure 1:**
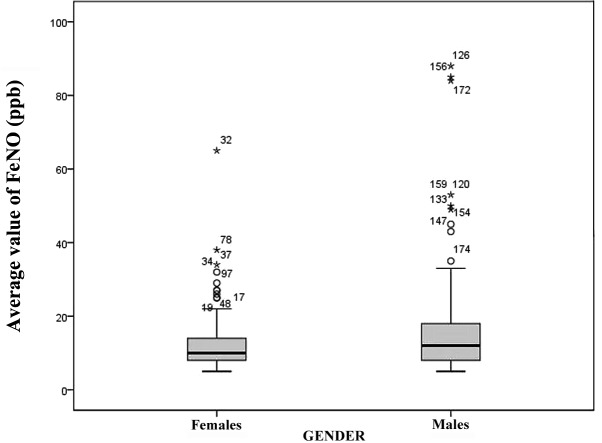
Comparison of FeNO between males and females. FeNO was significantly higher for males than females (P=0.007). FeNO, fractional exhaled nitric oxide.

The general characteristics of the participants in this study are displayed in Table I. The subjects with a FEV1 <80% were excluded, leaving 219 students available for analyses. Of the 219 Chinese individuals in the sample, 103 were males. The median FeNO value for the group was 11 ppb (IQR, 8–16 ppb). Table II shows the comparison of FeNO levels in children from other countries and areas. In males, the median FeNO was 13 with an IQR of 9–18, while in females the median was 10 with an IQR of 8–14. The FeNO level was significantly higher for males than females (P<0.05; Fig. 1).

**Figure 2:**
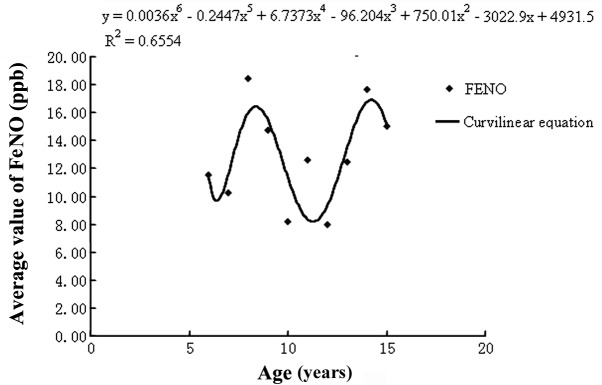
Correlation between age and FeNO. There was a significant correlation between age and FeNO level (R^2^=0.6554). FeNO, fractional exhaled nitric oxide.

**Figure 3:**
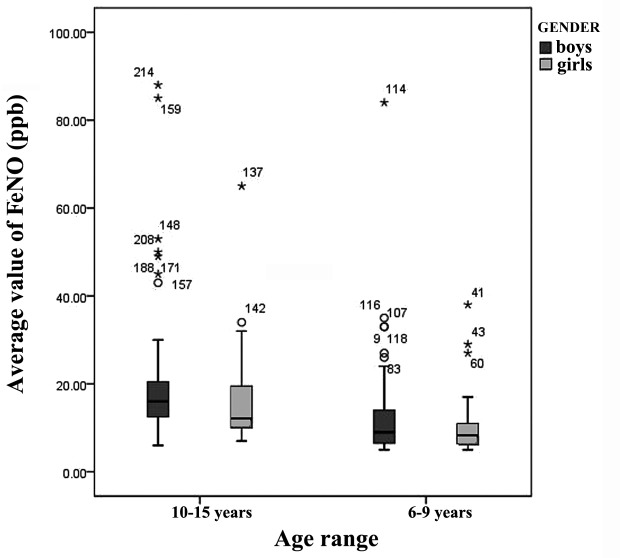
Median FeNO of males and females aged 6–9 years and 10–15 years. FeNO, fractional exhaled nitric oxide.

Furthermore, the correlation analyses revealed age to be correlated with FeNO levels (R^2^= 0.66). Other potentially significant factors, including height (R^2^= 0.14), weight (R^2^= 0.17), BMI (R^2^= 0.099), FVC_predicted_ (R^2^=0.037), FEV1_predicted_ (R^2^=0.023), FEV1/FVC_predicted_ (R^2^=0.02) and PEF (R^2^= 0.019) did not demonstrate a statistical correlation. We therefore identified reference FeNO values for northern Chinese children according to gender and age (Fig. 2). For individuals aged 6–9 years, the median FeNO level for males was 9 ppb (range, 6.5–14 ppb; 95% CI, 27.71 ppb) while for females it was 8 ppb (range, 6–11 ppb; 95% CI, 18.01 ppb). For individuals aged 10–15 years, the median FeNO level for males was 16 ppb (range, 12.75–20.25 ppb; 95% CI, 46.98 ppb) while for females it was 12 ppb (range, 10–19 ppb; 95% CI, 30.33 ppb; Fig. 3).

## Discussion

In the respiratory tract, NO is produced by a wide variety of cells, including airway epithelial cells, airway and circulatory endothelial cells and trafficking inflammatory cells, in large and peripheral airways and alveoli (13). NO is a gaseous signaling molecule that is generated by three isoenzymes of NO synthase (NOs) that are differentially regulated and expressed in the airways and that appear to play different pathophysiologic roles (14). The measurement of FeNO as a marker of airway inflammation is useful given the positive correlation with the degrees of airway hyper-responsiveness (AHR) (15) and eosinophilia in induced sputum, blood, bronchoalveolar lavage and bronchial mucosa. Asthma is accurately diagnosed in daily practice on the basis of subjective symptoms and FeNO levels, particularly in atopic patients (16,17).

Previous studies have suggested that FeNO is a valid and effective marker in the monitoring of treatment for pediatric asthma (18,19). However, it would be imprudent to recommend the systematic treatment of patients with high FeNO values and asthma with an allergy component who are asymptomatic or under good control (20). FeNO values have not been evaluated prospectively as an aid in asthma diagnosis; however, these measurements have been shown to contribute to optimal treatment and responsiveness to steroids (21) and may be used to distinguish asthma from other wheezing diseases (22). The values for FeNO in healthy children require investigation to develop the cut-off points for the judgement of normal vs. abnormal levels.

Since our study included healthy children from public school institutions, it was not feasible to measure atopy objectively by a skin-prick test or total specific immunoglobulin (Ig)-E. Therefore, we are not able to rule out the possibility that atopic subjects may have higher FeNO levels. However, a strict questionnaire was used to select subjects and we excluded those whose FEV1 was <80%; therefore, we consider our results to be reliable. In this study of a northern city in China, the median level of FeNO was 11 ppb (IQR, 8–16 ppb) and the FeNO was significantly associated with gender and age. Consequently, the FeNO levels were divided into four catetories, according to gender and age: males aged ≤9 years; males aged >9 years; females aged ≤9 years and females aged >9 years. Weight, height, BMI, FVC, FEV1, FVC/FEV1 and PEF were not correlated with FeNO levels.

A number of studies concerning FeNO in healthy children have been conducted and the conclusions of the current study are similar. However, these studies differ in the country of origin, sample ethnicity and time of study, as well as the number and ages of the individuals studied. While these data were not statistically analyzed, we compared them with ours to better understand the condition of FeNO in these children. In 2005 in Italy, Buchvald *et al* (2) reported a mean FeNO level of 9.7 ppb. In 2005 in Canada, Kovesi *et al* (23) reported a mean FeNO level for Caucasian Canadian children of 12.7 ppb (range, 11.8–13.7 ppb) and 22.8 ppb (range, 17.9–27.7 ppb) for Asian Canadian children aged 9.1–12.9 years. In 2010 in the United States, Perzanowski *et al* (1) suggested that the level of FeNO at age 7 is 12 ppb (range, 10.6–13.7 ppb). Two studies of FeNO in Hong Kong and Taiwan were conducted in 2005 and 2011 (4), respectively. Wong *et al* (3) reported that the level of FeNO for male children was 17 ppb (range, 10.7–36.6 ppb) and for female children was 10.8 (range, 7.8–17.6). Our results are consistent with the findings from the studies carried out in Hong Kong and Taiwan. This suggests that there are similarities in FeNO in these countries. Our findings are also in agreement with those from Caucasian Canadians but less so with those from Asian Canadians (24). We observed that the FeNO levels of Asian Americans are greater than those of Chinese individuals. Although observations have been made in individuals of the same ethnicity, different values for FeNO have been observed in different environments. Therefore, factors besides ethnicity may affect the value of FeNO in healthy children.

In FeNO studies of healthy children, a number of variables have been considered, including gender, age, weight, BMI, atopy and environmental tobacco smoke (ETS) (1,25,26). However, in adults, these factors are not correlated with FeNO (27).

Buchvald *et al* (2) observed a mean level of FeNO of 9.7 ppb and there was no difference in FeNO between males and females. FeNO was significantly and positively correlated with age in males and females, with an increase in FeNO of 5% per year (2). Although data remain equivocal, sensitization, rhinitis and conjunctivitis may be independent determinants of FeNO in subjects of all ages without asthma. However, the authors did not study the effect of ethnic background on FeNO (2). Multiple factors affect FeNO in healthy children, including race, age and height. One study used a formula for FeNO: Ln(FeNO) = 1.993 + age x 0.046 (4).

In the current study, gender and age were identified to be significant factors that affected FeNO in healthy children. Lung function is an important parameter in the diagnosis and monitoring of asthma (8). However, spirometry had no significant correlation with FeNO in healthy children or adults. Lung function indices, including FEV1-reversibility or provocation tests, are only indirectly associated with airway inflammation. FEV1 is ≥80% in a number of children whose airway clearance technique (ACT) is abnormal and whose FeNO is higher (28). Another study observed a correlation between FeNO and FEV1 while forced expiratory flow (FEF) did not independently contribute to FeNO variance (28). Official ATS clinical practice guidelines (21) recommend the use of FeNO in the monitoring of airway inflammation in patients with asthma and states that FeNO is a predictor of steroid responsiveness during the absence of induced sputum. An increasing number of countries and areas have participated in studies of FeNO and in the current study we examined more data to identify the factors that affect FeNO.

## Figures and Tables

**Table I:** General characteristics of subjects (n=219).

Parameter	Minimum	Maximum	Mean ± SD
Age (years)	6	15	10.16±2.805
Height (cm)	100	185	142.85±17.737
Weight (kg)	19	91	38.32±14.43
BMI (kg/m^2^)	9.4	30.5	18.085±3.1934
FVC_predicted_ (%)	69	130	88.59±9.881
FEV1_predicted_ (%)	80	140	96.17±10.475
FEV1/FVC_predicted_ (%)	71	119	105.42±6.972
PEF	36	122	74.26±18.365

SD, standard deviation; BMI, body mass index; FVC, forced vital capacity; FEV, forced expiratory volume; PEF, peak expiratory flow.

**Table II:** Comparison of FeNO levels in children from other countries and areas.

Study year	Author (ref)	Country/area (ethnicity)	N	Age range (years)	Mean FeNO (ppb)	Median FeNO (ppb)	FeNO IQR
2005	Buchvald *et al* (2)	Italy	405	4.0–17.0	9.7		
2005	Wong *et al* (3)	Hong Kong (Chinese)	253	11.0–18.0	25.3	17.0 (M)	10.7–36.6
					15.8	10.8 (F)	7.8–17.6
		Hong Kong (Caucasian)	33	11.0–18.0	14.9	11.6 (M)	8.2–19.3
						9.1 (F)	7.5–11.9
2008	Kovesi *et al* (23)	Canada (Caucasion)		9.1–12.9	12.7		11.8–13.7
		Canada (Asian)			22.8		17.9–27.7
2010	Perzanowski *et al* (1)	America		7		12	10.6–13.7
2011	Yao *et al* (4)	Taiwan			13.7		

Expiratory flow, 50 ml/sec for all subjects. M, male; F, female; IQR, interquartile range; FeNO, fractional exhaled nitric oxide.
